# In memoriam: Prof. Dr. rer. nat. Walter A. Maier (April 15, 1940 – March 19, 2026)

**DOI:** 10.1007/s00436-026-08779-y

**Published:** 2026-10-13

**Authors:** Helge Kampen, Günter A. Schaub

**Affiliations:** 1 Federal Research Institute for Animal Health, Friedrich-Loeffler-Institut, Südufer 10, Greifswald - Isle of Riems, 17493 Germany; 2 Zoology/Parasitology, Ruhr-University Bochum, Universitätsstr. 150, Bochum, 44780 Germany

**Figure:**
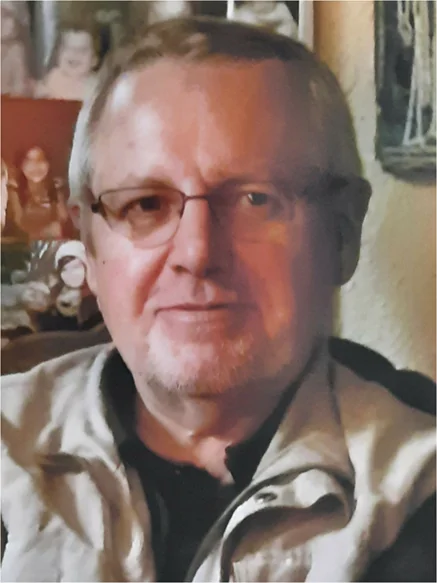

On March 19, 2026, Prof. Dr. Walter Maier, a German medical entomologist and parasitologist, passed away after several years of severe illness.

Walter Maier was born on July 15, 1940 in Landsberg am Lech, southern Germany. Immediately after the end of World War II – his father having been killed in action in 1944 – his mother moved with him and his sister into emergency housing in Hüfingen, where he started school. While still in primary school, the family moved again to live with his grandparents in Baden-Baden where Walter Maier completed his primary education and transferred to a ‘Gymnasium’ (German academic secondary school). From 1954, he attended the upper level of high school in Bregenz, Austria, graduating with ‘Matura’ (Austrian high school certificate) in 1959.

To pursue his keen interest in the natural sciences, he enrolled that same year at the Technical University of Karlsruhe to study chemistry, botany, and geography. From 1961 to 1967, he continued his university studies at the Albert-Ludwigs-University of Freiburg, focusing on biology, chemistry, limnology, and geology. He completed his studies with a diploma thesis entitled “Species-specific differences in protein patterns (particularly enzymes) in the larval hemolymph of selected species of the genus *Chironomus* (Meig.)” under the supervision of Prof. Dr. Wolfgang Wülker. He married Gudrun in 1966, and their daughter Heidrun was born in 1968. Continuing his work under Prof. Wülker, Walter Maier served as a research associate in the German Research Foundation’s priority programme “Parasite Physiology” from 1968 to 1970, and held a position as a research assistant at the Institute of Biology I (Zoology) of the University of Freiburg from 1970 to 1971. During this time, he worked on and completed his doctoral thesis, entitled “The hemolymph of *Chironomus* (Dipt.) and its alteration by parasitic mermithids.”

That same year, following the birth of his second daughter Isabel, Walter Maier moved with his family to Bonn, after having been offered a research position at the Institute of Medical Parasitology of Bonn’s University Hospital by the institute’s director, Prof. Dr. Gerhard Piekarski. He began working as a research assistant in 1973 with the aim of a habilitation (postdoctoral lecturing qualification). In 1981, under the new director of the institute, Prof. Dr. Hanns Martin Seitz, he achieved his habilitation with the thesis “On the effects of *Plasmodium* infection on the vector and sporozoite formation (using the examples of *Plasmodium cathemerium* – *Culex pipiens fatigans* and *P. berghei* – *Anopheles stephensi*).” Subsequently, Walter Maier was granted the venia legendi (authorization to teach) by the University of Bonn’s Faculty of Medicine in the field of Medical Parasitology and was appointed an associate professor in 1988. As he held a permanent position at the institute and both he and his family were happy in the Bonn area, Walter Maier remained at the institute until his retirement in 2005.

Walter Maier’s scientific work focused primarily on pathogens transmitted by blood-feeding arthropods, the vectors themselves, and the interactions between these, their vertebrate hosts and the environment (Maier 1976, 2002; Chutmongkonkul et al. 1992; Maier et al. 2003). He worked with malaria parasites and mosquitoes, borreliae and ticks, and numerous other endo- and ectoparasites, while also addressing the chemotherapy of toxoplasmosis and malaria (Maier and Piekarski 1976; Kaiser and Maier 2002). In addition to his research activities, he was deeply involved in the institute’s diagnostic tasks regarding parasites and arthropods and undertook extensive teaching duties for students of medicine, pharmacy, and biology. His four-week, full-day practical courses ‘Medical Entomology’ (summer semester) and ‘Medical Parasitology’ (winter semester) attracted great interest among advanced biology students and served as a key source for recruiting students for their diploma and doctoral theses in biology. By the time he retired, he had supervised 118 state examination, diploma, and doctoral theses across the fields of biology, medicine, and pharmacy.

Walter Maier tirelessly championed and promoted the field of medical entomology in both scientific circles and the public. Following German reunification, he intensified contacts with colleagues in East Germany and, in 1993, co-founded the ‘Medical Arachno-Entomology’ working group, which was affiliated to the German Society for Parasitology and the German Society for General and Applied Entomology. By uniting medical-entomological expertise from east and west, Walter Maier fostered collaborations that have remained fruitful to this day and contributed to the reunification process. The working group’s annual meetings have a focus on the practical taxonomic identification of selected groups of vectors and pests which make it unique in the field. Eleven years later, Walter Maier proposed transforming the working group into an independent scientific society: the German Society for Medical Entomology and Acarology. For many years, he served as the chairperson of this society which attracted not only scientists but also pest control professionals from across the German-speaking world, extending well beyond Germany’s borders. Even after his retirement, Walter Maier remained an enthusiastic participant in the meetings, engaging in lively discussions with colleagues and students alike.

Walter Maier not only succeeded in inspiring students for his field of expertise, but was also highly respected and exceptionally well-connected, both nationally and internationally. He maintained close academic ties with colleagues in Great Britain, France, Egypt, Kenya, Cameroon, Japan, Thailand, and the Philippines. Furthermore, he provided advice to physicians, government agencies, ministries, staff from the embassies then based in Bonn, and the World Health Organization (e.g., Karanis et al. 1993; Hofmann et al. 2000; Maier 2000).

In the 1980s, many university positions previously held by experts in medical parasitology and entomology were filled by molecular biologists and geneticists. Academic profiles like the one Walter Maier presented became scarce, and the field of classical medical entomology was no longer viewed as innovative or worthy of funding, causing a shortage of comprehensively trained young scientists. Walter Maier persevered undeterred, supporting his doctoral students in every way possible. It is thanks to him that the field of medical entomology survived a lean period in Germany, a time when vector-borne diseases were considered of little relevance in Central Europe. In recent years, however, the field has gained renewed interest and is once again becoming a matter of urgency: vectors are spreading and the number of associated cases of disease is rising – driven largely by globalization and global warming. In Germany, particular concern surrounds the Asian tiger mosquito, a highly efficient potential vector of numerous life-threatening viruses now established in many regions, as well as *Culex* mosquitoes that are able to transmit West Nile virus. Walter Maier was no longer active when these new challenges which require well-founded expertise in medical entomology arose, but thanks to him, a small number of specialists have remained in Germany who can apply their expertise to managing the problems and pass their knowledge on still another generation of scientists.

After retiring, Walter Maier spent much of his time at his forest cabin, devoting himself to his hobby of painting. He also enjoyed the company of his five grandchildren. In his final years, a severe illness imposed significant limitations and hardships on both him and his family.

In Walter Maier, we have lost one of the last broadly trained German medical entomologists, as well as a valued colleague, a dedicated mentor, and a good friend. We extend our deepest sympathy to his family.

## Data Availability

No datasets were generated or analysed during the current study.
